# Enological Suitability of Indigenous Yeast Strains for ‘Verdejo’ Wine Production

**DOI:** 10.3390/foods12091888

**Published:** 2023-05-04

**Authors:** Jennifer Vázquez, Ana Maria Mislata, Victor Vendrell, Carlos Moro, Sergi de Lamo, Raúl Ferrer-Gallego, Imma Andorrà

**Affiliations:** 1 VITEC, Wine Technology Centre, 43730 Falset, Tarragona, Spain; anamaria.mislata@vitec.wine (A.M.M.); sergi.delamo@vitec.wine (S.d.L.); raul.ferrer@vitec.wine (R.F.-G.);; 2 Bodega Emina Rueda (Bodega Matarromera, S.L.), Ctra. Medina del Campo-Olmedo. Km 1.4, 47400 Medina del Campo, Valladolid, Spain; vvendrell@matarromera.es (V.V.);

**Keywords:** indigenous yeast selection, *Saccharomyces cerevisiae*, *Metschnikowia pulcherrima*, alcoholic fermentation, verdejo wine, volatile compounds, sensory evaluation

## Abstract

The use of indigenous yeasts for the production of wines is a tool to defend the typicity of a particular region. The selection of appropriate indigenous yeasts ensures the maintenance of oenological characteristics by simulating spontaneous alcoholic fermentation (AF) while avoiding the risks of stuck or sluggish fermentations. In this study, autochthonous yeasts from Verdejo grape juice (Appellation of Origin Rueda) were selected, identified, and characterized to exploit the characteristics of the ‘terroir’. The fermentation capacity of seven strains was studied individually at the laboratory scale. The most suitable strains (*Saccharomyces cerevisiae*: *Sacch 1*, *Sacch 2*, *Sacch 4,* and *Sacch 6*) and *Sacch 6* co-inoculated with *Metschnikowia pulcherrima* were characterized at the pilot scale. The fermentation kinetics, bioproduct release, volatile composition, and sensory profile of the wines were evaluated. Significant differences were found, especially in the aroma profile. In particular, *Sacch 6* and *Sacch 6* co-inoculated with *M. pulcherrima* produced higher amounts of ethyl esters and acetates and lower amounts of higher alcohols than the spontaneous AF. Wines inoculated with indigenous yeasts had higher sensory scores for fruit aromas and overall rating. The selection of indigenous yeasts improved the aroma of Verdejo wines and could contribute to determining the wine typicity of the wine region.

## 1. Introduction

Wines produced by spontaneous fermentation result from complicated biochemical reactions where microorganisms such as yeasts, bacteria, and fungi play a decisive role. During traditional winemaking, there is a sequential succession of yeasts engaged in the alcoholic fermentation (AF) process. A large part of the important secondary metabolites and aromatic compounds responsible of the final wine quality and complexity are released during AF [1,2,3].

Yeast microbiota diversity and its evolution during the AF are influenced by several factors including geographical location, climatic conditions, agronomical practices, and anthropogenic and biotic factors, among others. The grape variety, the harvest time, the health of the grapes, the age of the vineyard, and the vinification conditions are also important aspects to be considered [4,5]. The predominant non-*Saccharomyces* species found on grapes and during the early stages of fermentations are *Candida*, *Metschnikowia*, *Hanseniaspora*, *Kluyveromyces*, *Pichia*, *Hansenula*, *Rhodotorula*, *Brettanomyces,* and *Cryptotocus* [6]. At the beginning of the AF process, non-*Saccharomyces* yeasts can play a substantial role until approximately mid-fermentation, when *S. cerevisiae* ends up colonizing the wine AF [6,7,8,9]. As a result, *S. cerevisiae* has simply been considered as the wine yeast par excellence and it has been selected and optimized for commercial use as starter culture. The primary rationale of wineries for inoculation with commercial yeast is to produce wines with a uniform quality during different years, thereby avoiding the variability associated with spontaneous AF and the risk of spoilage [10,11]. However, the use of this practice can influence the natural microflora of musts and often leads to its removal. Likewise, wines produced by the inoculation of commercial strains usually show low variability, complexity, and typicity. Regarding this fact, the selection of autochthonous yeast to be used as starter cultures has been described as a suitable microbiological alternative to mimic spontaneous AF, offering wines with the distinctive sensory qualities from a specific region [12,13].

Obtaining a yeast collection of oenological interest from a specific area implies knowledge of the ‘*terroir*’ biodiversity, including evaluating the microbiota present in grapes and during the spontaneous AF with the subsequent oenological characterization of each strain which is isolated. In addition, recovering the biodiversity of wine ecosystems, as native strains are better adapted to the climatic conditions of the region, allows us to use these yeast strains as starter cultures, ensuring reproducible and predictable controlled fermentations [14]. Moreover, the yeast selection procedure can be adapted to acquire strains which are able to improve the wine quality and its complexity [15].

As mentioned before, the strains used during winemaking are responsible for the presence or absence of some flavors and other non-volatile compounds related with desirable sensory perception. Interesting wild yeasts have been described as being able to release compounds and metabolites to improve the wine quality by various mechanisms such as the metabolism of the grape’s sugar and nitrogen components, the enzymatic hydrolysis of grape aroma precursors, flavor, and, finally, by the yeast cell autolysis [3,16]. For instance, *Torulaspora delbrueckii* and *M. pulcherrima* are able to generate lower levels of volatile acidity than *S. cerevisiae* [3,17]. Furthermore, *M. pulcherrima* and *Hanseniaspora uvarum* are great producers of esters which enhance the aromatic composition of wines [18,19]. *Starmerella bacillaris* can produce high levels of glycerol [3] and *Lachancea thermotolerans* can improve lactic acid production [20]. Exploiting the abilities of these yeasts in mixed cultures (by sequential or co-inoculated fermentations) can positively contribute to wine flavor and other characteristics [21,22].

Rueda became the first recognized Appellation of Origin in the autonomous community of Castilla y Leon (Spain). Verdejo is a native white variety with wide international recognition for white wines with intense fruit and tropical aromas, and a balanced acidity. However, limited studies have been undertaken to improve its oenological properties using indigenous strains, even though the study of the yeast biodiversity of Verdejo grapes can lead to a suitable selection of indigenous yeasts.

In the present research, relevant information of the indigenous flora for a fit production of Verdejo white wines by spontaneous fermentations is reported. The fermentations took place in controlled laboratory- and pilot-scale conditions. Finally, the wines were differentiated by evaluating their chemical composition and sensorial analysis.

## 2. Materials and Methods

### 2.1. Yeast Strains

**Figure A1:**
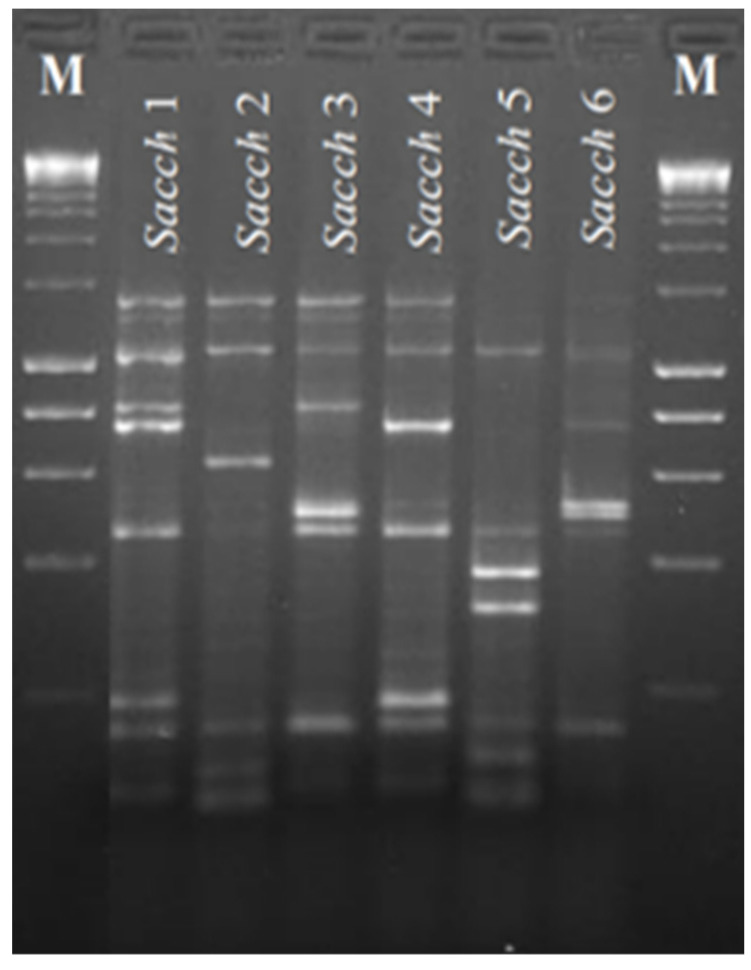
Electrophoretic patterns of inter-delta region obtained from six *Saccharomyces cerevisiae* randomly isolated at the middle and in the end of spontaneous alcoholic fermentation of Verdejo grapes. Lines 1 and 8 correspond with 1 Kb DNA Ladder (M), lines 2 to 7 correspond to strains from *Sacch 1* to *Sacch 6*.

**Figure A2:**
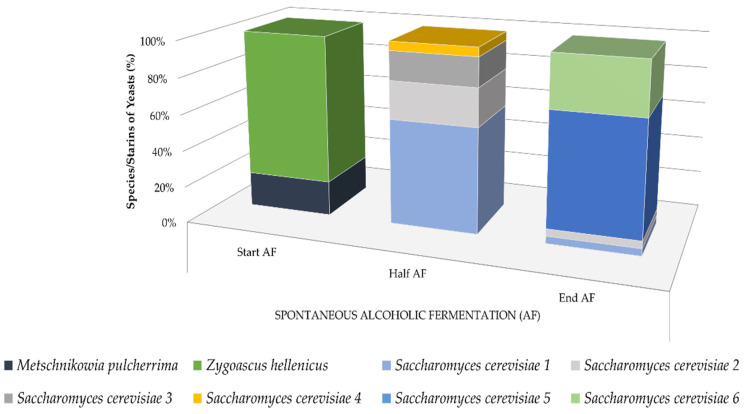
The diversity of yeast at different stages of spontaneous alcoholic fermentation (AF).

Six *S. cerevisiae* (*Sacch 1*, *Sacch 2*, *Sacch 3*, *Sacch 4*, *Sacch 5,* and *Sacch 6* (CECT 13191)) and one *M. pulcherrima* (*Mp*; Genebank access code EU037994.1) yeast strains were isolated from spontaneously fermented grape must, originating from Verdejo grapes from ‘La Bodeguilla’ vineyard (X: 348,257.65; Y: 4,593,529.34) at Bodegas Matarromera S.A, in Valbuena del Duero (Spain) located within the Rueda Appellation of Origin. Briefly, at the beginning, middle, and end of the spontaneous AF, samples were taken and were spread on yeast peptone dextrose agar (YPDA). At the end of the incubation period (48 h at 28 °C), 24 yeast colonies with a distinct morphological appearance were selected and purified. All selected strains were identified by PCR-RFLP analysis of 5.8S-ITS rDNA [23]. The colonies identified as *S. cerevisiae* were typified at strain level by the analysis of inter-delta regions [24] (Figure A1, Appendix A). PCR products of non-*Saccharomyes* strains were digested with the restriction endonucleases *Cfo*I, *Hae*III, and *Hin*fI. Yeasts were identified to species level by comparison of the amplified product and their restriction fragments sizes with the sizes described by Zarzoso et al., 1999. One strain of each different profile obtained were selected for amplifying the D1/D2 domain encoding the subunit 26s rRNA [25], followed by sequencing. Yeast diversity during the spontaneous AF is represented in Figure A2 (Appendix B).

### 2.2. Grape Must

Musts were obtained from Verdejo grapes harvested in 2016 and 2017 vintages. Grapes were hand-picked, put into 15 kg boxes, and transported (at 4 °C) to the experimental winery of VITEC (Wine Technology Center, Tarragona, Spain). Grapes were then destemmed, crushed, and transferred into 50 L steel tanks. The cold settling was carried out with 1.2 g/hL of Lysozim (Laffort^®^, Floirac, France) at 16 °C for 24 h. The basic chemical composition of the must was as follows: brix grade 20.0 ± 0.0, density 1082.3 ± 1.2 g/L, total sulfur dioxide 1 ± 0 mg/L, free sulfur dioxide 1 ± 0 mg/L, total sugar 190.3 ± 1.3 g/L, nitrogen assimilable by yeast 217 ± 1 mg/L, primary amino nitrogen 134.5 ± 1.0 mg/L, pH 3.4 ± 0.0, ammonia 100 ± 5 mg/L, total acidity 5.2 ± 0.0 g/L expressed as tartaric acid, acetic acid 0.10 ± 0.00 g/L, malic acid 2.9 ± 0.1 mg/L, and lactic acid 0.1 ± 0.0 g/L.

### 2.3. Characterization of Yeasts Fermentative Performances

To characterize the fermentative capacity of the seven yeast isolates (*Sacch 1*, *Sacch 2*, *Sacch 3*, *Sacch 4*, *Sacch 5*, *Sacch 6,* and *Mp*), laboratory-scale fermentations were carried out in triplicate in 500 mL erlenmeyer flasks containing 220 mL of Verdejo grape juice. Cultures of the seven yeast isolates were pre-incubated in 15 mL of yeast-peptone-dextrose (YPD) medium at 25 °C in a rotatory shaker (100 rpm) for 48 h to obtain an inoculum size of 10^7^–10^8^ cells/mL. From these pre-cultures, Verdejo must flasks were inoculated to obtain a final yeast density of 2 × 10^6^ cells/mL and incubated at 17 °C without agitation. The fermentation process was monitored though density measurement until sugar concentrations were lower than 2 g/L. Samples were taken for every flask during fermentation process to analyze total cells by counting in Thoma’s chamber and viable cells by plating in YPD agar. The imposition of the inoculated yeast strains was evaluated at the end of AF following the methods described previously.

### 2.4. Pilot Scale Vinifications with the Selected Yeast Strains

Based on the results obtained from laboratory scale characterization, the most suitable yeast strains were selected and inoculated (2 × 10^6^ cells/mL) from pre-cultures (10^7^–10^8^ cells/mL) in stainless steel tanks containing 30 L of Verdejo grape juice. Fermentations with each of the four selected *S. cerevisiae* strains were conducted (*Sacch 1*, *Sacch 2*, *Sacch 4,* and *Sacch 6*). Moreover, one mixed fermentation was carried out with *Sacch 6* and *M. pulcherrima* (10% and 90%, respectively, of the total initial inoculum population). Furthermore, one spontaneous fermentation was carried out as a control. Each fermentation was performed in duplicate at controlled temperature (17 °C, during the entire process). As described previously, fermentations were monitored by classical analytical techniques (density, total and viable cells). Once AF was completed (<2 g/L of residual sugars), samples were taken and spread on YPDA. As described before, 24 colonies were selected to evaluate the prevalence of the inoculated yeast strains using the analysis of inter-delta regions [24]. The obtained wines were clarified with the addition of 40 g/hL bentonite Microcol^®^ (Laffort^®^, Floirac, France), cold-stabilized at 4 °C for 5 days, and filtered through 1 and 0.45 µm pore sizes. Finally, the stabilized wines were bottled.

### 2.5. Oenological Parameters Analysis

The oenological parameters, including the content of glucose-fructose, density, alcohol content, pH, total acidity concentration expressed as tartaric acid (ATT), volatile acidity concentration (expressed as acetic acid), total and free sulfur dioxide, glycerol, and malic, lactic, and acetic acids, were measured in all wines (laboratory and pilot scale) according to the methods recommended by the Compendium of International Methods of Analysis—Organization of Vine and Wine (OIV) 2020 [26].

### 2.6. Determination of Wine Aroma Compounds

The volatile aroma compounds of all pilot-scale vinifications were analyzed by gas-chromatography GC 7890A (Agilent Technologies, Santa Clara, CA, USA) coupled to a 5975C MSD inert mass spectrometry 5975C MSD (Electronic Shock Source Triple Axis Detector) according to Mislata et al., 2020 [27]. Briefly, the volatile compounds of base wines were extracted using SPME (DVB/CAR/PDMS). The column was a DB-WAX UI (60 m × 0.25 mm × 0.25 µm, Agilent Tech). A constant flow of 2.1 mL/min of helium was used as carrier gas. The results of the volatile compounds were semi-quantitative data in relation to the response provided by the internal standard (2-octanol). All analyses were performed in duplicate.

### 2.7. Organoleptic Evaluation

The quantitative descriptive analysis (QDA) was performed by a trained tasting panel following the normative ISO 8586:2012. Thus, a total of six wines (Wild AF, *Sacch 1, Sacch 2, Sacch 4, Sacch 6,* and *Sacch 6 + Mp*) were tasted by 10 judges in the normalized ISO 8589:2007 room of VITEC. Wine sensory evaluation was classified into different attributes, including color (intensity and evolution), aroma (intensity and profile), flavor (sourness, unctuosity, bitterness, persistence, burning, and reduction), and global punctuation. Among the aroma profile, fruity aromas (tropical, citric, and candied fruit aromas), spicy, floral, chemical, and vegetal were considered as interesting attributes. Panelists were required to rate the intensity of the wine parameters using a five-point scale (1 = absence, 5 = maximum intensity). All of the obtained data were analyzed by FIZZ software (Biosystems, V.2.47B, Barcelona, Spain).

### 2.8. Statistical Analysis

The data were subjected to a one-way analysis of variance (ANOVA) and Tukey’s post-hoc test to evaluate the effect of each fermentation. The results were considered statistically significant at a *p*-value less than 0.05 (XLSTAT statistical and data analysis solution software, Lumivero, V.2023.1.4, Denver, USA). A Principal Component Analysis (PCA) was performed to visualize a 2D plot of the first two principal components (PCs) using XLSTAT statistical and data analysis solution software (Lumivero, V.2023.1.4, Denver, USA).

## 3. Results

### 3.1. Fermentative Potential of Yeast Isolates

**Figure 1:**
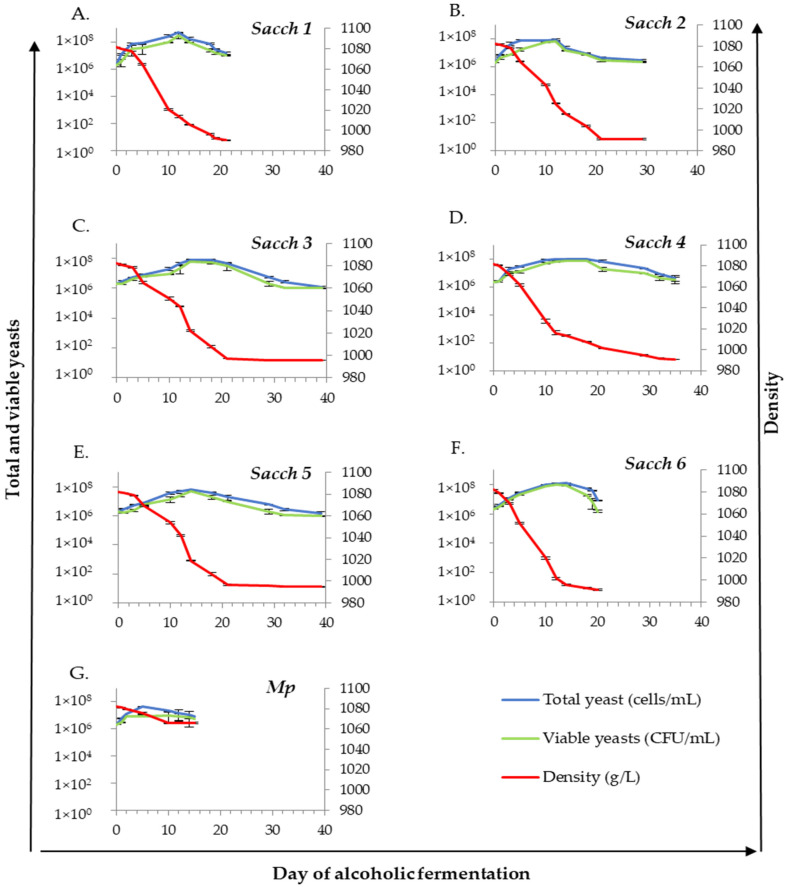
The evolution of the seven laboratory-scale fermentations obtained by inoculating six indigenous *Saccharomyces cerevisiae* strains (*Sacch*) and one indigenous strain of *Metschnikowia pulcherrima* (*Mp*). Errors bars represent SD of *n* = 2.

The fermentation kinetics of the indigenous yeast isolates and the oenological parameters of the wines studied at the laboratory scale are shown in Figure 1 and Table 1, respectively. All *S. cerevisiae* yeasts (Figure 1A–F) exhibited a high fermentative capacity when compared with the *M. pulcherrima* strain, which was not able to consume the sugar from the grape juice (Figure 1G). The total yeast cell concentration of *M. pulcherrima* increased to above 4 × 10^7^ cells/mL five days after the fermentation began. The density decreased from 1082 to 1066 g/L in 10 days and, subsequently, remained constant. Thus, after five days with constant density values, it was determined as stuck fermentation.

Among the isolated *S. cerevisiae* yeast strains, all were able to prevail at 100% at the end of fermentation. *Sacch 6* and *Sacch 1* showed the maximum AF efficiency, reaching 10^8^ cells/mL after 12 days and ending the AF in 20 and 21 days (Figure 1A,F), respectively. The residual sugar for both *Sacchs* was <2 g/L (Table 1). Although *Sacch 2* and *Sacch 4* reached similar cell populations after 12 days (9 × 10^7^ cells/mL), *Sacch 2* required 29 days to reduce sugars to below 2 g/L, whereas *Sacch 4* took 35 days to complete the AF (Figure 1B,D). Finally, the treatments inoculated with *Sacch 3* and *Sacch 5* increased the total cell population during the first 14 days (6.6 × 10^7^ and 8.2 × 10^7^ cells/mL, respectively) and, after 40 days, they had relatively higher residual sugar contents (5 g/L) (Figure 1C,E).

The physico-chemical properties of wines for ‘Verdejo,’ established for the governing rules of PDO Rueda, are as follow: alcoholic grade > 1.5 vol.%, acetic acid ≤ 0.65 g/L, total SO_2_ ≤ 180 mg/L, and total sugar expressed as fructose and glucose ≤ 4 g/L. Thus, the laboratory-scale fermentations produced by the selected strains *Sacch 1*, *Sacch 2*, *Sacch 4,* and *Sacch 6* showed good fermentation kinetics and appropriate oenological parameters to satisfy the PDO rules. In contrast, *Sacch 3* and *Sacch 5* showed high values of residual sugar and *Sacch 3* showed a lower alcohol content than 11.5 vol.%. Both *Sacch 3* and *Sacch 5* did not accomplish the PDO Rueda standards and, therefore, they were not selected to perform pilot-scale fermentations.

### 3.2. Evolution of Density and Yeast Population at Pilot Scale Fermentations

**Figure 2:**
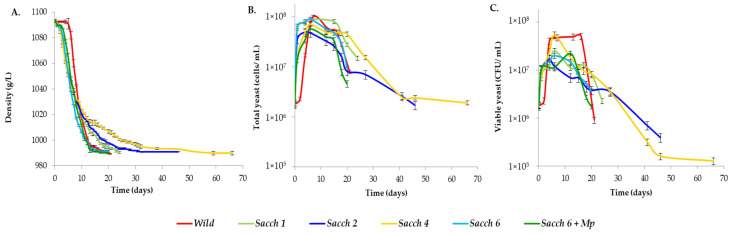
The monitoring of the (**A**) density, (**B**) total yeasts (expressed in cells/mL), and (**C**) viable yeasts, expressed in CFU (colony forming unit) at pilot-scale fermentations. Spontaneous AF (Wild), four single fermentations with selected strains of *S. cerevisiae* (*Sacch 1*, *Sacch 2*, *Sacch 4* and *Sacch 6*) and one mixed fermentation with *S. cerevisiae* and *M. pulcherrima* strains (*Sacch 6 + Mp*). Errors bars represent SD of *n* = 2.

The fermentations at the pilot scale were performed by the inoculation of pure cultures using the selected strains (*Sacch 1*, *Sacch 2*, *Sacch 4*, and *Sacch 6*) and one mixed culture of *Sacch 6 + Mp*. Spontaneous fermentation by wild yeast (Wild) was used as control. The density and the yeast content (total and viable cells) were monitored during the fermentations (Figure 2). The imposition of *S. cerevisiae* isolates was 100% at the end of all of the fermentations. Spontaneous AF (Wild) slowly started to ferment after five days (Figure 2A), achieving the highest population (1.1 × 10^8^ cells/mL) at day 7, and its fermentation finished with a population of 8.7 × 10^6^ cells/mL and 1.0 × 10^6^ CFU/mL after 21 days (Figure 2B,C).

All of the yeast isolates completed AF with reduced sugar levels < 2 g/L (Table 2). However, differences in the fermentation kinetics were found depending on the yeast inoculate which was used (Figure 2). *Sacch 6* and the mixed fermentation (*Sacch 6 + Mp*) were the fastest to consume the sugars of the must (20 days) (Figure 2A). Wild AF also consumed sugars rapidly, in only 22 days. In general, the yeast population increased during the first seven days and, after that, underwent a reduction (Figure 2B,C). The final cell viability of *Sacch 6* AF was the highest (3.9 × 10^6^ CFU/mL).

Conversely, *Sacch 1*, *Sacch 2,* and *Sacch 4* were the slowest fermentations, ending at 24, 46, and 66 days, respectively (Figure 2A). When the fermentations ended, the amount of yeast biomass of *Sacch 1* showed a higher cell density population (1.7 × 10^7^ cells/mL) and more viable cells (2.5 × 10^6^ CFU/mL) than spontaneous AF, whereas the total yeast populations of the *Sacch 2* and *Sacch 4* fermentations dramatically decreased until reaching 2 × 10^6^ cells/mL, approximately. The cell viability also decreased in both of the fermentations, being 4.3 × 10^5^ CFU/mL in the case of *Sacch 2* and 1.4 × 10^5^ CFU/mL in the case of *Sacch 4*.

### 3.3. Basic Parameters and Volatile Composition of Wines

The results of the basic chemical composition analysis of the final wines are shown in Table 2. Significant differences were found between the wines. All of the wines obtained by indigenous strains showed a higher relation of free SO_2_/total SO_2_ and lower values of total acidity than the values from spontaneous fermentation. Regarding the acetic acid production, the low content of this acid produced by *Sacch 6 + Mp* wines (0.07 g/L) should be noted. Moreover, *Sacch 6 + Mp* also showed the lowest levels of residual sugar. The wines obtained from spontaneous fermentation showed higher alcohol and glycerol concentrations, followed by the wines inoculated with *Sacch 6 + Mp* and *Sacch 6*.

The detailed results of the aromatic compounds are presented in Table 2. A total of 18 aromatic compounds were quantified and grouped into the four most abundant families according to their chemical structure (esters, acetates, higher alcohols, and fatty acids).

The most abundant and representative family of fermentative aromas are esters. These compounds present aromatic descriptors related to fruits and flowers [28,29,30]), giving the wines fresh and fruity notes. As observed in Table 2, the total content of esters did not present significant differences between wines. However, it is necessary to highlight that the wines obtained from *Sacch 6* and *Sacch 6 + Mp* presented the highest values. When analyzing the compounds individually, ethyl hexanoate, ethyl octanoate, and ethyl decanoate in all cases exceeded their odor threshold, providing the wines with aromatic notes of green apple, pineapple, pear, and grape (Table A1) [28,31]. In addition, all of them presented the highest concentration in the fermentations made by *Sach 6*, and *Sacch 6 + Mp*.

In the family of acetates, the total composition did present significant differences, especially *Sacch 6* and *Sacch 6 + Mp*, which showed significantly higher levels, mainly due to the high concentrations of ethyl acetate, isoamyl acetate, and 2-phenyethylacetate. These last two compounds (isoamyl acetate and 2-phenylacetate) presented values that were well above their odor threshold, providing aromatic notes related to banana and flowers, respectively (Table A1) [31]. Some acetates, depending on their concentration, can provide pleasant or unpleasant notes to wines, such as the ethyl acetate. When this compound appears in low concentrations, it provides positive notes such as sweet fruits, but when it is found in high concentrations it can cause undesirable aromas, such as glue or solvent. In general, none of the obtained wines exceeded the odor threshold of ethyl acetate [32], indicating the absence of defects.

The family of alcohols is characterized by presenting aromatic descriptors related mainly to flowers [33,34,35]. The total concentrations (Table 2) presented significant differences, showing the highest values for Wild yeast followed by *Sacch 6* and *Sacch 6 + Mp*. In general, although none of the samples exceeded the odor threshold (Table A1) for the four compounds studied, it should be noted that the high alcohol concentrations were mainly due to the isoamyl alcohol and 2-phenylethyl alcohol. Furthermore, although all of the inoculated wines decreased the amount of isobutanol, it is important to highlight that *Sacch 6* and *Sacch 6 + Mp* reduced its concentration by 67% and 73%, respectively, when compared to the Wild fermentations. This compound at high concentrations usually contributes unpleasant aromas of solvents and glue [31].

Finally, the family of fatty acids presented significant values in terms of total concentration, with *Sacch 6* and *Sacch 6 + Mp* showing the highest values. In general, fatty acids characteristically contribute aromatic notes of soap, cheese, and rancid to wines at high concentrations (Table A1) [31]. However, it should be noted that none of the compounds presented values above their odor threshold in any of the studied samples.

**Figure 3:**
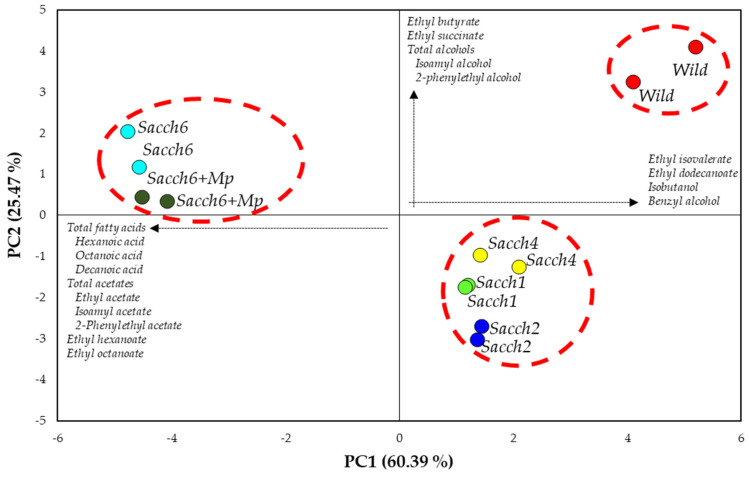
Biplot of principal components analysis (PCA; 85.86% of variance) using the volatile aroma compounds as variables. PC1 explains 60.39% of variance: (+); ethyl isovaleriate, ethyl dodecanoate, isobutanol, and benzyl alcohol. (-); Total fatty acids, hexanoic, octanoic and decanoic acids, total acetates, ethyl, isoamyl and 2-phenylethyl acetate, and ethyl hexanoate and ethyl octanoate. PC2 explains 25.47% of variance: (+); ethyl butyrate and ethyl succinate, total alcohols, isoamyl alcohol, and 2-phenylethyl alcohol.

Principal component analysis (PCA) was applied to correlate the different variables of volatile aroma compounds and highlight some grouping patterns within the different final wines produced by the different yeast isolates (Figure 3). PCA explained 85.86% of the variability within the wine samples. The first component (PC1) accounted for 60.39%, whereas PC2 explained an additional 25.47% of the variability. Spontaneous fermentations (control wines) were positioned in the positive direction of PC1 and PC2 and were associated with higher concentrations of esters such as ethyl butyrate, ethyl succinate, ethyl isovalerate, and ethyl dodecanoate. The wines inoculated with different indigenous yeast isolates were differentiated into two groups, and both were characterized by a lower prevalence of higher alcohols than the control group. The *Sacch 6* and *Sacch 6 + Mp* group, located in the negative axis of PC1, differed by presenting the highest content of fatty acids and acetate compounds.

**Figure 4:**
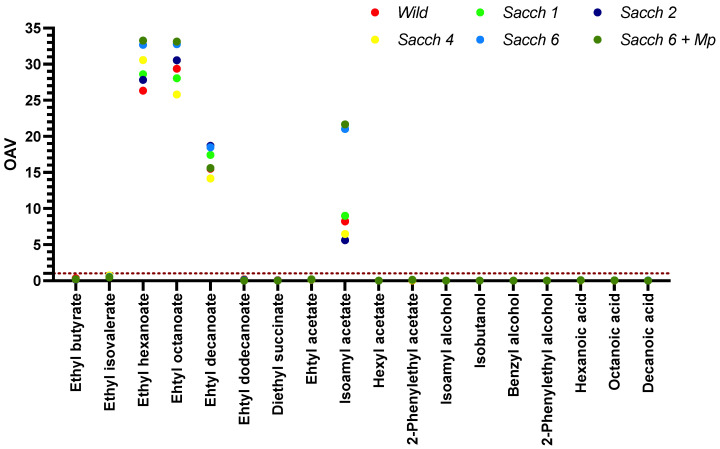
Odorant active values (OAV) of the 18 volatile aroma compounds of the wines obtained by spontaneous alcoholic fermentation (*Wild*), the four single fermentations inoculated with *S. cerevisiae* (*Sacch 1*, *Sacch 2*, *Sacch 4* and *Sacch 6*) and the mixed fermentation inoculated with *S. cerevisiae* and *M. pulcherrima* strains (*Sacch 6 + Mp*).

The contribution of the volatiles to the aroma of the wine was evaluated for each compound through the odor activity values (OAV). This parameter is calculated as the ratio between the concentration of each compound and its corresponding perception threshold [36,37]. If the calculated OAV for certain compound results greater than the unity, this compound can be considered as an active aroma [27,32]. Figure 4 shows the OAV for the 18 volatile compounds for which odor thresholds were available. In general, a total of four compounds were active aromas (ethyl hexanoate, ethyl octanoate, ethyl decanoate, and isoamyl acetate). *Sacch 6* and *Sacch 6 + Mp* showed the highest OAV values of ethyl hexanoate and ethyl octanoate, as well as isoamyl acetate, contributing fruity notes such as pineapple, pear, and banana to these wines.

### 3.4. Sensory Profiles of Final Wines

**Figure 5:**
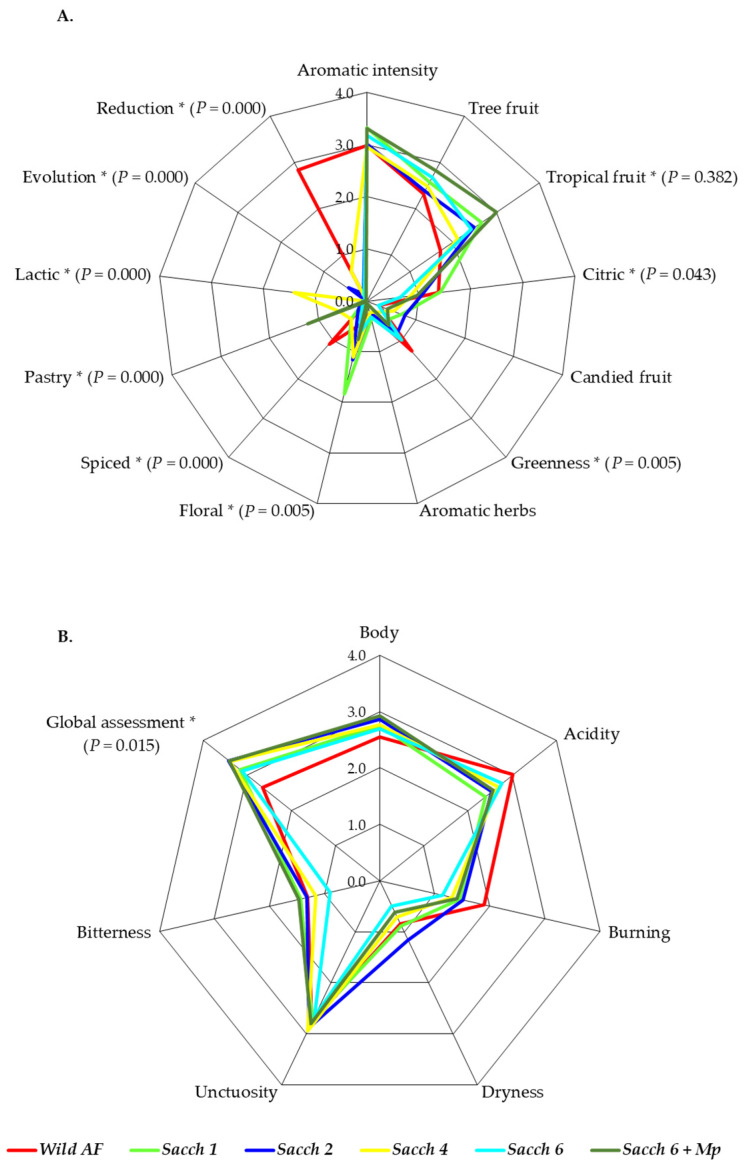
Radar chart of aromatic profiles (**A**), and test aspect and global assessment (**B**), of the wines obtained by spontaneous alcoholic fermentation (Wild), the four single fermentations inoculated with *S. cerevisiae* (*Sacch 1*, *Sacch 2*, *Sacch 4* and *Sacch 6*) and the mixed fermentation inoculated with *S. cerevisiae* and *M. pulcherrima* strains (*Sacch 6 + Mp*). Results obtained by the mean and standard deviation of the scores given by the testers. The asterisk symbol indicates statistically significant differences (*p* < 0.5) between different wines.

The aromatic profile of the six Verdejo wines obtained in this study was characterized by its aromatic intensity, with notes of aromatic tropical fruit such as pineapple and banana, notes of citrus such as orange and grapefruit, and touches of floral aroma (Figure 5A). Nevertheless, the analysis of variance exhibited significant differences for nine of the wine descriptors. The wines which were fermented spontaneously showed the highest values of greenness, spiced, and reduction. In contrast, they were the ones with the lowest values of tropical fruit and pastry descriptors. The highest scores for the floral descriptor were shown in *Sacch 1* wines. In comparison, it was shown that the wines obtained with the isolate *Sacch 2* were the most evolved sensorially. The wines inoculated with *Sacch 4* showed the highest score for the lactic descriptor. The wines inoculated with *Sacch 6* were quite balanced due to not standing out regarding any attribute. Finally, the highest scores of pastry and tropical fruit descriptors were observed in the wines obtained by the mixed inoculation (*Sacch 6 + Mp*). No significant differences were observed in the sensorial evaluation of generic attributes; nevertheless, the tasters highly appreciated all of the wines produced by indigenous yeast isolates, with the spontaneously fermented wines the worst valued (Figure 5B).

## 4. Discussion

In recent years, there has been a strong interest driven by wine industry demand for wines with distinctive chemical and sensory properties to study the possibility of using specific indigenous yeast strains with the aim to obtain starter cultures that are potentially well adapted to a definite grape must, exploiting the biodiversity of a specific ‘terroir.’ In the present study, seven yeast isolates from Verdejo plots in the Designation of Origin Rueda were characterized in order to obtain a final wine with enhanced organoleptic properties. The approach involved the isolation and selection of the indigenous strains from spontaneous fermentation and their subsequent inoculation to assess the influence of the strains on fermentation performance and on the final wine characters.

The results which were obtained confirmed the applicability of these tools to increase the wine yeast diversity of Verdejo wines with suitable oenological properties. Yeast characterization was undertaken to assess the influence of the yeast strain on the fermentation performance. The results of laboratory-scale fermentations are in accordance with the observations generally reported in the literature for wine yeast, showing that *S. cerevisiae* strains possess higher fermentative power than non-*Saccharomyces* strains [6,38,39,40,41,42]. *M. pulcherrima*, when isolated, showed low fermentative capacity, probably due to its sensibility to high concentrations of ethanol, making the mixed use with *S. cerevisiae* to completely ferment the grape must necessary [21,38,43]. Although *S. cerevisiae* generally possess the ability required to perform an efficient fermentation, its ability is strain-dependent due to *Sacch 3* and *Sacch 5* needing to be discarded because they were unable to consume all of the sugar of the must after 40 days.

*Sacch 1*, *Sacch 2*, *Sacch 4,* and *Sacch 6* were considered suitable yeast strains to carry out pilot-scale fermentations. Although the fermentative behavior was appropriate for all of the indigenous strains tested, the winemaking scale clearly influenced the *Sacch 2* and *Sacch 4* strains, considerably increasing the duration of fermentation and the loss of cell viability. An appropriate wine yeast starter, in addition to avoiding sluggish and stuck fermentations and enhancing the wine character [14], must achieve rapid fermentation, which implies significant time and cost savings and, thus, the optimization of the process for the winery. Regarding this fact, *Sacch 6* and mixed fermentation (*Sacch 6 + Mp*) followed by spontaneous fermentation and, finally, *Sacch 1*, were the fastest in consuming sugars. However, spontaneous fermentation took longer to start consuming sugars. This slowdown might be related to the diversity of wild yeasts at the beginning of spontaneous fermentation, where various species which only tolerate low levels of alcohol dominate for the first days. When the alcohol level reaches a higher amount, those species begin to die off and alcohol-tolerant *S. cerevisiae* (present on grape in a very low proportion) dominate the fermentation process. This fact could leave the grape must more vulnerable to infection by additional spoilage microorganisms and oxidation [40,44,45]. Regarding microvinifications inoculated with *Sacch 6* as monoculture and those co-inoculated with *M. pulcherrima*, differences were not observed between their fermentation performances. It has been described that *M. pulcherrima* can cause fermentation delays due to the production of pulcherrimin, which has a killing effect against many yeasts, including *S. cerevisiae* [46]. However, this phenomenon depends on the different biotypes within the *M. pulcherrmia* species [47].

The selection of final wines was based on the evaluation of fermentation by-products (residual sugar, glycerol, acetic acid, SO_2_, etc.) and aroma and flavor compounds such as ethyl esters, acetates, higher alcohols, and fatty acids. These compounds have been reported to vary based on yeast species and among yeast strains [18,48,49,50]. The wines from yeast isolates (*Sacch 1*, *Sacch 2*, *Sacch 4*, *Sacch 6*, and *Sacch 6 + Mp)* showed more favorable ratios between free and total SO_2_ than the spontaneous fermentation wines. SO_2_ binds very strongly to acetaldehyde and microorganisms. Thus, a high proportion of free SO_2_ can be an indicator of a lower risk of oxidation and microbial spoilage, leading to a wider margin of action as regards any subsequent addition of the additive [51,52,53].

The spontaneous fermentations, *Sacch 6* and *Sacch 6 + Mp*, resulted in wines with relatively higher ethanol concentrations. As previously mentioned, wines produced by *Sacch 6* and by co-inoculation with *Sacch 6* and *M. pulcherrima* showed similar fermentative performances. In addition, they also showed similar final ethanol concentrations. The respiro-fermentative regulatory mechanisms of *M. pulcherrima* have been exploited to reduce ethanol concentrations in combined fermentations with *S. cerevisiae* [21,54,55,56]. However, suitable results are dependent on the time and cell proportion inoculation. Our results might indicate that sequential inoculation is generally more favorable than simultaneous inoculation, to allow the expression of non-*Saccharomyces* properties [19,57].

Glycerol is the main polyol synthesized by yeasts during AF [58]. It is a colorless, odorless, and non-volatile compound that provides unctuousness to wines. The spontaneous wines followed by mixed (*Sacch 6 + Mp*) and *Sacch 6* microvinifications were the ones that generated higher amounts of glycerol. As described by Ruiz et al., 2018 [59], *S. cerevisiae* co-inoculated with *M. pulcherrima* generates a higher glycerol content than single inoculations with *S. cerevisiae*.

The total acidity of the wines obtained was in the range of 4.6 and 5.9 g/L, with the values of the wines which were spontaneously fermented being the highest. The total acidity is one of the differentiators which are responsible for the harmonized taste of the wine. In the finished beverage, this value should not exceed 10 g/L; however, the most desirable value is between 4 and 6 g/L [60]. Acetic acid is the main component of volatile acidity, and is normally present in wines in low concentrations (0.20–0.60 g/L). The obtained results indicate that concentrations of acetic acid can vary depending on the strain. *Sacch 6* and mixed fermentations (*Sacch 6 + Mp*) produced the lowest amounts of acetic acid. Furthermore, *M. pulcherrima* contributes to a lower production of acid acetic in wine [11,17,61]. Low concentrations of this compound provide a pleasant acidity in the mouth and serve as a precursor to acetate esters, which are responsible for the fruity character in many wines.

From the results, it appears that the isolated yeast affected the amount of volatile compounds in the wines. However, the results found during the calculation of the smell activity (OAV) of each compound indicate that only ethyl hexanoate, ethyl octanoate, ethyl decanoate, and isoamyl acetate participate in the overall aroma of the Verdejo wines, and these will probably be the most perceived by the human nose [32]. Nevertheless, it is important to highlight the ability of volatile compounds to interact between one another and with other compounds present in the wine, which may result in different flavors than those expected from individual compounds [62,63].

Our findings indicate that the wines fermented with indigenous yeast inoculum showed a higher score in their fruity character due to a lower final concentration of higher alcohols and a higher concentration of specific fruity esters and acetates. Ethyl esters and acetates are generally considered to have a positive influence on wine aroma by contributing fruity and floral characteristics [64,65]. The highest perception of the fruity aroma descriptor by the tasting panel was for the wines co-inoculated with *M. pulcherrima*, which agrees with the highest values of compounds related with fruity aroma odors such as ethyl hexanoate, ethyl octanoate, and isoamyl acetate [49]. *M. pulcherrima* is well known to increase the total concentration of esters with ethyl octanoate being the most relevant [19]. The fermentations involving *M. pulcherrima* increased the ethyl octanoate by around 11%, 15%, 8%, and 22%, compared with the spontaneous fermentations, *Sacch 1* and *Sacch 2*, respectively. These increments are in accordance with other works such as Dutraive et al., 2019 [66], which reports increments around 14%. However, when comparing the *Sacch 6 + Mp* with the *Sacch 6* fermentation, ethyl octanoate only increased around 1%. In fact, *Sacch 6* was able to increase other compounds such as ethyl hexanoate, ethyl decanoate, and isoamyl acetate, indicating that this indigenous strain may be considered as a suitable starter to produce desirable aroma compounds. The relation of the higher amounts of these compounds with the presence of one or more additional enzymes responsible for synthesized and hydrolyzed flavor-enhanced compounds [11,67] could be an important target for future research.

The lowest perception of the fruity aroma descriptor was for the wines which were spontaneously fermented. These wines, in addition to showing low values of ethyl hexanoate, ethyl octanoate, ethyl decanoate, and isoamyl acetate, were characterized by their higher content of isoamyl alcohol and isobutanol. Although the higher alcohol concentrations were at optimal levels (<300 mg/L) to add a desirable level to complexity [68], it has been described that ethanol can suppress the fruit aroma attributes [69]. In addition, volatile fatty acids were found at sub-sensory threshold levels in all of the wines produced in this research, with the wines obtained by indigenous yeasts showing the highest amounts of these compounds. Without being above the fatty acid odor threshold, these compounds can impart the wines with fruity notes and can improve their complexity [49]. It is important to highlight that SO_2_ also improves the taste and retains the wine fruity flavors and freshness of aroma [70]. As mentioned before, wines from inoculated yeasts showed higher values of SO_2_ than spontaneous AF.

## 5. Conclusions

The selection study allowed us to obtain a general vision of the indigenous yeast present in Verdejo wines. Selecting strains can be used as starters in wine fermentation when the oenological parameters remain at appropriate level. According to the results found in this study, tasters successfully differentiated the wines produced from indigenous yeast isolates, which justified the wines having better organoleptic and chemical characteristics than those produced using spontaneous fermentation. The fermentation kinetics were better for *Sacch 6*, *Sacch 6* co-inoculated with *M. pulcherrima*, and *Sacch 1*. Moreover, the wines produced from *Sacch 6* and *Sacch 6 + Mp* resulted in an improved aromatic profile and in a low volatile acidity production.

As a result, the indigenous *S. cerevisiae* strain (*Sacch 6*) was selected in order to be developed as a Verdejo wine starter culture at the industrial level (Emina Rueda cellar of Bodegas Matarromera, S.A.). The results obtained from the implantation studies carried out with *Sacch 6* allowed us to confirm its suitability for oenological use, evidencing that the proposed yeast selection methodology was appropriate. In fact, with this research, Emina Rueda cellar has launched on the market its first wine produced by selected yeast isolated from its own vineyard.

## Figures and Tables

**Table 1:** Basic oenological parameters of the wines fermented at laboratory scale with different indigenous yeasts isolated.

Yeast Strain	Total SO_2_ (mg/L)	Alcoholic Strength (vol.%)	Residual Sugar (g/L)	Acetic Acid (g/L)	Glycerol (g/L)
*Sacch 1*	5.0 ± 2.0 ^d^	11.80 ± 0.06 ^bc^	1.5 ± 0.6 ^bc^	0.32 ± 0.02 ^c^	4.6 ± 0.2 ^a^
*Sacch 2*	9.0 ± 1.0 ^c^	11.93 ± 0.04 ^ab^	1.9 ± 0.0 ^b^	0.38 ± 0.01 ^b^	4.5 ± 0.3 ^a^
*Sacch 3*	24.0 ± 1.0 ^a^	11.02 ± 0.07 ^e^	5.1 ± 0.1 ^a^	0.56 ± 0.01 ^a^	4.3 ± 0.2 ^a^
*Sacch 4*	11.0 ± 1.0 ^bc^	11.75 ± 0.04 ^cd^	1.6 ± 0.1 ^b^	0.55 ± 0.03 ^a^	4.4 ± 0.1 ^a^
*Sacch 5*	13.0 ± 1.0 ^b^	11.66 ± 0.07 ^d^	5.0 ± 0.2 ^a^	0.53 ± 0.02 ^a^	4.6 ± 0.2 ^a^
*Sacch 6*	5.0 ± 1.0 ^d^	12.04 ± 0.02 ^a^	0.8 ± 0.7 ^c^	0.20 ± 0.00 ^d^	4.6 ± 0.1 ^a^

Different letters in superscript indicate significant differences among different yeast isolates for each studied parameter expressed as the mean value and SD (standard deviation) of *n* = 2 by ANOVA and Tukey HSD post-test (*p* < 0.05).

**Table 2:** The content of chemical and volatile compounds in final wines fermented spontaneously (Wild), using five different starters of *S. cerevisiae (Sacch 1*, *Sacch 2*, *Sacch 4*, and *Sacch 6)* and one mixed starter with *S. cerevisiae* and *M. pulcherrima* (*Sacch 6 + Mp*).

Compounds	Wild	*Sacch 1*	*Sacch 2*	*Sacch 4*	*Sacch 6*	*Sacch 6 + Mp*
Basic parameters						
Total SO_2_ (mg/L)	65 ± 2 ^b^	87 ± 2 ^a^	80 ± 2 ^a^	66 ± 5 ^b^	85 ± 3 ^a^	80 ± 3 ^a^
Free SO_2_ (mg/L)	20 ± 1 ^c^	34 ± 3 ^ab^	39 ± 1 ^a^	27 ± 2 b^c^	31 ± 2 ^ab^	32 ± 2 ^ab^
Free SO_2_/Total SO_2_	0.31 ± 0.01 ^a^	0.39 ± 0.03 ^b^	0.48 ± 0.03 ^c^	0.41 ± 0.00 ^b^	0.36 ± 0.02 ^d^	0.40 ± 0.01 ^b^
Alcoholic grade (vol.%)	13.55 ± 0.03 ^a^	13.30 ± 0.07 ^bc^	13.21 ± 0.06 ^c^	13.20 ± 0.01 ^c^	13.42 ± 0.02 ^ab^	13.44 ± 0.02 ^ab^
Residual sugar (g/L)	0.4 ± 0.0 b^c^	1.3 ± 0.7 ^ab^	1.8 ± 0.0 ^a^	1.6 ± 0.1 ^a^	0.9 ± 0.0 ^abc^	0.1 ± 0.0 ^c^
pH	3.44 ± 0.09 ^a^	3.52 ± 0.10 ^a^	3.63 ± 0.10 ^a^	3.56 ± 0.10 ^a^	3.6 ± 0.10 ^a^	3.6 ± 0.10 ^a^
ATT (g/L)	5.9 ± 0.1 ^a^	5.1 ± 0.1 ^ab^	4.7 ± 0.4 ^b^	4.6 ± 0.3 ^b^	5 ± 0.0 ^b^	4.9 ± 0.0 ^b^
Acetic acid (g/L)	0.18 ± 0.00 ^d^	0.48 ± 0.00 ^a^	0.41 ± 0.00 ^c^	0.44 ± 0.00 ^b^	0.12 ± 0.00 ^e^	0.07 ± 0.00 ^f^
Malic acid (g/L)	3.20 ± 0.00 ^a^	2.60 ± 0.00 ^b^	2.60 ± 0.00 ^b^	1.50 ± 0.00 ^e^	2.40 ± 0.00 ^c^	2.10 ± 0.00 ^d^
Lactic acid (g/L)	0.1 ± 0.0 ^b^	0 ± 0.0 ^bc^	0.1 ± 0.0 ^b^	0.7 ± 0.0 ^a^	0 ± 0.0 ^c^	0 ± 0.0 ^c^
Glycerol (g/L)	5.7 ± 0.0 ^a^	4.2 ± 0.0 ^e^	3.8 ± 0.0 ^f^	4.4 ± 0.0 ^d^	5.1 ± 0.0 ^c^	5.3 ± 0.0 ^b^
Total esters (µg/L)	27,415.7 ± 423.7 ^a^	27,543.1 ± 33.2 ^a^	29,490.4 ± 3127.5 ^a^	24,676.1 ± 2968.2 ^a^	31,042.8 ± 1151.9 ^a^	29,810.4 ± 1284.3 ^a^
Ethyl butyrate	142.4 ± 7.1 ^a^	58.0 ± 0.6 ^c^	53.9 ± 6.7 ^c^	76.8 ± 8.3 ^bc^	89.1 ± 7.8 ^b^	85.7 ± 1.8 ^b^
Ethyl isovalerate	2.0 ± 0.2 ^a^	1.8 ± 0.1 ^ab^	1.5 ± 0.2 ^b^	2.2 ± 0.1 ^a^	1.6 ± 0.2 ^b^	1.4 ± 0.1 ^b^
Ehyl hexanoate	2105.6 ± 52.2 ^e^	2288.4 ± 48.5 ^cd^	2224.9 ± 38.8 ^de^	2444.5 ± 63.6 ^bc^	2604.6 ± 21.1 ^ab^	2659.9 ± 21.8 ^a^
Ehtyl octanoate	17,033.3 ± 22.5 ^ab^	16,266.2 ± 254.2 ^ab^	17,707.9 ± 1652.64 ^ab^	14,954.4 ± 1780.7 ^b^	19,001.9 ± 415.2 ^ab^	19,214.6 ± 696.5 ^a^
Ethyl decanoate	7759.3 ± 512.8 ^a^	8715.7 ± 184.1 ^a^	9335.5 ± 1501.8 ^a^	7082.9 ± 1248.1 ^a^	9236.5 ± 740.6 ^a^	7796.7 ± 561.6 ^a^
Ethyl dodecanoate	285.6 ± 11.2 ^a^	190.7 ± 10.6 ^b^	146.3 ± 18.5 ^b^	92.7 ± 10.6 ^c^	71.3 ± 9.2 ^cd^	29.8 ± 2.2 ^d^
Diethyl succinate	87.2 ± 18.3 ^a^	22.2 ± 0.5 ^b^	20.2 ± 0.3 ^b^	22.5 ± 0.9 ^b^	27.7 ± 0.1 ^b^	22.4 ± 0.5 ^b^
Total acetates (µg/L)	2255.2 ± 467.0 ^b^	2434.1 ± 26.1 ^b^	1829.1 ± 143.5 ^b^	2132.5 ± 241.4 ^b^	4950.6 ± 358.5 ^a^	5068.8 ± 38.2 ^a^
Ethyl acetate	884.9 ± 127.5 ^ab^	917.8 ± 13.3 ^ab^	878.3 ± 84.6 ^b^	1045.6 ± 186.9 ^ab^	1358.3 ± 164.2 ^a^	1359.2 ± 34.8 ^a^
Isoamyl acetate	1316.4 ± 329.6 ^b^	1436.8 ± 38.4 ^b^	896.4 ± 59.4 ^b^	1033.6 ± 55.8 ^b^	3364.3 ± 193.1 ^a^	3465.1 ± 6.8 ^a^
Hexyl acetate	13.5 ± 2.2 ^a^	11.4 ± 0.1 ^a^	12.1 ± 1.0 ^a^	11.8 ± 1.9 ^a^	12.0 ± 0.3 ^a^	10.9 ± 1.1 ^a^
2-Phenylethyl acetate	40.5 ± 7.9 ^d^	68.1 ± 1.1 ^c^	42.3 ± 1.5 ^d^	41.6 ± 3.2 ^d^	216.1 ± 1.0 ^b^	233.6 ± 4.4 ^a^
Total alcohols (µg/L)	1762.1 ± 49.2 ^a^	1125.9 ± 8.6 ^cd^	1001.9 ± 57.4 ^d^	1227.4 ± 34.5 ^bc^	1376.8 ± 56.0 ^b^	1233.7 ± 2.8 ^bc^
Isoamyl alcohol	1275.4 ± 33.9 ^a^	749.1 ± 8.4 ^bc^	661.2 ± 49.7 ^c^	863.9 ± 73.3 ^b^	893.2 ± 54.5 ^b^	786.9 ± 8.6 ^bc^
Isobutanol	58.7 ± 2.7 ^a^	35.9 ± 0.1 ^c^	41.2 ± 4.6 ^bc^	48.8 ± 4.9 ^ab^	18.9 ± 1.3 ^d^	16.1 ± 0.9 ^d^
Benzyl alcohol	3.7 ± 0.2 ^a^	3.1 ± 0.1 ^ab^	3.0 ± 0.2 ^ab^	3.4 ± 0.5 ^ab^	3.0 ± 0.1 ^ab^	2.6 ± 0.1 ^b^
2-Phenylethyl alcohol	424.2 ± 12.3 ^a^	337.8 ± 0.4 ^b^	296.6 ± 3.3 ^b^	311.2 ± 43.2 ^b^	461.7 ± 0.3 ^a^	428.1 ± 6.7 ^a^
Total fatty acids (µg/L)	591.0 ± 9.1 ^c^	686.7 ± 6.8 ^c^	657.9 ± 0.9 ^c^	697.2 ± 70.5 ^c^	987.6 ± 3.5 ^a^	843.6 ± 19.3 ^b^
Hexanoic acid	143.6 ± 1.0 ^c^	167.1 ± 0.1 ^b^	155.9 ± 7.8 ^bc^	176.5 ± 11.6 ^b^	227.6 ± 0.5 ^a^	217.6 ± 1.4 ^a^
Octanoic acid	367.7 ± 6.7 ^c^	410.5 ± 6.9 ^c^	400.8 ± 3.1 ^c^	416.9 ± 41.3 ^c^	605.0 ± 0.1 ^a^	521.3 ± 9.3 ^b^
Decanoic acid	79.8 ± 3.3 ^b^	109.1 ± 0.1 ^b^	101.2 ± 3.8 ^b^	103.8 ± 17.6 ^b^	154.9 ± 3.0 ^a^	104.6 ± 8.5 ^b^

Different letters in superscript indicate significant differences between treatments (fermentations) for each studied parameter expressed as the mean value and SD (standard deviation) of *n* = 2 by ANOVA and Tukey HSD post-test (*p* < 0.05).

## Data Availability

No new data were created or analyzed in this study. Data sharing is not applicable to this article.

## Appendix B

**Table A1:** Families of the individual volatile compounds, the corresponding aromatic descriptors, and odor thresholds in model wine solution.

Families & Compounds	Descriptor	Odor Threshold (µg/L)
Esters		
Ethyl butyrate	Pineapple, strawberry	400 (d)
Ethyl hexanoate	Fruit, green apple, anise	80 (d)
Ethyl octanoate	Pineapple, pear, floral, sweet	580 (a)
Ethyl decanoate	Fruit, grape, nice	500(a)
Ethyl dodecanoate	Floral, fruit, sweet, cream	1500 (c)
Ethyl isovaleriate	Fruit, apple	3 (d;e)
Diethyl succinate	Fruit, melon	1200 (a)
Alcohols		
Isoamyl alcohol	Flowers, solvent	60,000 (a)
Isobutanol	Flowers, nail polish	75,000 (a)
Benzyl alcohol	Citrus, sweet	200,000 (b)
2-phenylethyl alcohol	Roses, pollen, perfume	200,000 (a)
Acetates		
Ethyl acetate	Sweet fruit, pinapple, solvent, balsamic	7500 (f)
Isoamyl acetate	Banana, sweet	160 (a)
Hexyl acetate	Pear, apple, cherry	670 (a)
2-phenylethyl acetate	Green tea, fruit, flowery	1800 (a)
Fatty Acids		
Hexanoic acid	Soapy, cheese	3000 (a)
Octanoic acid	Soapy, rancid	10000 (a)
Decanoic acid	Soapy, rancid	6000 (a)

(a) Peinado, R.A. et al., 2004 [31]; (b) Gómez-Míguez, M.J. et al., 2007 [35]; (c) Yong-Sheng, T. et al., 2009 [29]; (d) Ferreira, V. et al., 2000 [28]; (e) Francis, I.L. et al., 2005 [30]. (f) Guth, H. et al., 1997 [32].
